# Relative Dose Intensity of Trabectedin and Outcome of Advanced L‐Sarcomas

**DOI:** 10.1002/cam4.71131

**Published:** 2025-08-19

**Authors:** Stephen Poitureau, Marie‐Cécile Le Deley, Mehdi Brahmi, Emmanuelle Bompas, Jean‐Emmanuel Kurtz, Simon Nannini, Christophe Perrin, Loïc Lebellec, Thomas Ryckewaert, Redha Ould‐Ammar, Clémence Leguillette, Jean‐Yves Blay, Nicolas Penel

**Affiliations:** ^1^ Lille University, Medical School Lille France; ^2^ Department of Clinical Research and Innovation Centre Oscar‐Lambret Lille France; ^3^ Université Paris‐Sud, UVSQ, CESP, INSERM, Université Paris‐Saclay Villejuif France; ^4^ Department of Medical Oncology Centre Léon Bérard, University Claude Bernard Lyon I Lyon France; ^5^ Department of Medical Oncology Institut de Cancérologie de L'ouest Nantes France; ^6^ Department of Medical Oncology Institut de Cancérologie de Strasbourg‐Europe Strasbourg France; ^7^ Medical Oncology Unit, Centre Eugène Marquis Rennes France; ^8^ Department of Medical Oncology Centre Oscar Lambret Lille France; ^9^ Department of Clinical Research and Innovation Centre Oscar Lambret Lille France; ^10^ Department of Medical Oncology Centre Oscar‐Lambret Lille France; ^11^ ULR 2694 – Metrics: Evaluation Des Technologies de santé et Des Pratiques médicales, CHU of Lille University of Lille Lille France

**Keywords:** dose reduction, relative dose intensity, soft tissue sarcoma, toxicity, trabectedin

## Abstract

**Background:**

Trabectedin, which is approved for advanced soft tissue sarcoma management, has a complex mechanism of action, but can be classified as an alkylating agent. The need to maintain a high relative dose intensity (RDI) is not clearly established in this clinical setting.

**Methods:**

We conducted a retrospective study in five expert centers to compare the progression‐free survival (PFS) and overall survival (OS) of patients with advanced L‐Sarcomas (liposarcomas or leiomyosarcoma) according to the RDI calculated over the first three cycles (RDI < 80% and RDI ≥ 80%). Comparisons of patients' characteristics were done using Chi‐2, Fisher exact, and Wilcoxon tests. Associations between PFS/OS and RDI were estimated and tested in Cox models.

**Results:**

Out of 332 patients treated with trabectedin between 09/1999 and 12/2021, 244 have received at least 3 cycles before progression. Among these 244 patients, the median RDI during the first 3 cycles was 83% (range, 48%–106%), the mean RDI was 81% (±14%) and 106 patients had RDI < 80%. An RDI < 80% was more frequently observed in patients treated in a center with a high volume of activity (82/169, 49%, vs. 24/75, 32%, *p* = 0.02), in patients who had previously received pazopanib (12/18, 67%, vs. 94/225, 42%, *p* = 0.04), and in patients who experienced grade 3 neutropenia during the first cycle (56/77, 73% vs. 35/127, 28%, *p* < 0.001). PFS did not significantly differ according to RDI (*p* = 0.08): HR_(< 80%/≥ 80%)_ = 0.79 (95% CI, 0.61–1.03), median PFS = 8.4 months (7.0–9.3) when RDI < 80% vs. 5.9 months (4.4–6.8) when RDI ≥ 80%. We observed no significant difference in terms of OS (*p* = 0.53): HR_(< 80%/≥ 80%)_ = 0.92 (95% CI, 0.70–1.20), median OS = 18.2 months (15.6–23.4) when RDI < 80% vs. 15.8 months (13.2–19.7) when RDI ≥ 80%.

**Conclusion:**

This retrospective study does not support a link between high trabectedin RDI and PFS or OS for advanced L‐sarcoma patients.

## 1 Introduction

Soft tissue sarcomas (STS) constitute a heterogeneous group of rare cancers, representing about 1% of all adult solid cancers [1]. STS are ubiquitous, since they develop from mesenchymal cells in various body sites [2]. The most common primary sites are the limbs (especially the lower extremity), followed by the trunk and the retroperitoneum [3]. As of today, there are more than 100 different STS histological subtypes [4]. Among the most common are leiomyosarcoma (LMS) and liposarcoma (LPS), commonly abbreviated as L‐sarcomas [5].

[6]. For the treatment of localized disease, surgical resection in a reference center is the cornerstone of curative therapy. Although there have been progress in local therapy and management through standardized approaches in expert centers, distant metastasis will develop in 50% of patients diagnosed with STS [7].

The overall prognosis for patients with locally advanced or metastatic STS is poor, with an estimated median overall survival under 20 months [8, 9]. In 2024, anthracycline‐based chemotherapy remains the first line of palliative treatment of advanced/metastatic STS [8, 10, 11]. Doxorubicin is the most commonly used anthracycline. In second‐line, there are different available options, including pazopanib for non‐adipocytic STS [12], eribulin for LPS [13], ifosfamide [14], and gemcitabine with dacarbazine or docetaxel [15, 16]. The treatment may depend on the histological subtype.

Among these therapies, trabectedin, a marine‐derived drug, acts as an alkylating agent but also affects other key cell biology processes in the tumor and its microenvironment (it has selective anti‐inflammatory, immunomodulatory and anti‐angiogenic properties) [17, 18, 19, 20, 21, 22, 23, 24]. It was approved for the treatment of adults with advanced soft tissue sarcoma, after failure of anthracycline and ifosfamide, or for those who are unsuited to receive these agents. It was approved by the European Medical Agency (EMA) in 2007, by the U.S. Food and Drug Administration (FDA) in 2015, following the results of a pivotal, randomized phase III trial in patients with advanced LPS or LMS (L‐sarcomas) [25]. In France, trabectedin has had approval (AMM) since 2017; however, the use of trabectedin is restricted to labeled centers.

Among all STS histological subtypes, L‐sarcomas, especially myxoid/round cell liposarcoma, are regarded as the most sensitive to trabectedin [26, 27]. L‐sarcomas were the subtypes studied in the pivotal study of Demetri et al. [25]

The different phase I–II studies found that trabectedin can be administered at the dose of 1.5 mg/m^2^ every 3 weeks, with a tolerable toxicity [19, 28]. Moreover, this toxicity was manageable in phase III and IV studies, especially by adding corticosteroids [25, 29, 30, 31]. The T‐Dis study led to two main findings: first, that trabectedin should not be discontinued after six cycles if there is a disease control; and secondly, that there is no cumulative toxicity with the trabectedin regimen [32, 33]. Nonetheless, dose reduction or cycle delay during the course of treatment with trabectedin occurs in more than a third of the patients, which leads to a reduction of the dose intensity of trabectedin [9, 25]. The most common adverse events that result in reduction of dose intensity are hematological and hepatic toxicities.

However, two different hypotheses provide the foundation for maintaining a high dose of chemotherapy: the Norton‐Simon hypothesis and the Goldie‐Coldman hypothesis [34, 35, 36, 37]. The importance of dose intensity has been shown in multiple studies, especially in hematological malignancies and breast cancers [38, 39, 40, 41]. Dose intensity can be measured with the relative dose intensity (RDI) that is, the amount of drug administered per unit of time expressed as the fraction of that used in the standard regimen (dose per unit body surface area per unit time [mg/m2 per week]) [42]. Finally, most studies have shown that an RDI > 80%–85% correlates with better outcomes in various cancers [39, 40, 43].

In phase III studies, the median trabectedin relative dose intensity ranged between 80 to 90% in STS [9, 44]. However, in a phase IV prospective study, reflecting real‐life management of patients, authors found that the median relative dose intensity was about 75% [29]. As the dose intensity is a critical parameter explaining the efficacy of conventional cytotoxic agents, especially alkylating agents, and given that trabectedin acts as an alkylating agent, there is a room to assess the association between trabectedin dose intensity and its efficacy.

Consequently, we carried out an observational retrospective study in five labeled sarcomas centers in France to assess the relation between the relative dose intensity and its efficacy in locally advanced/metastatic L‐sarcomas management.

## 2 Patients and Methods

### 2.1 Study Design and Objectives

This is a multicenter retrospective study conducted in five expert centers from the NETSARC network (network of expert centers labeled by the French National Cancer Institute). The primary objective was to study the association between the relative dose intensity (RDI) of trabectedin, calculated over the first 3 cycles, and the clinical efficacy in terms of progression‐free survival (PFS) according to RECIST in patients who received at least 3 cycles of treatment with trabectedin before progression. The secondary objectives were to study the association between RDI calculated over the first 2 cycles and PFS, the association between RDI calculated over the first 3 cycles and overall survival (OS), the association between RDI and objective response (according to RECIST) at first evaluation, the association between toxicity observed during the first cycle and RDI over the first 3 cycles, and to describe the patient and tumor characteristics.

### 2.2 Eligibility Criteria

The inclusion criteria were: (i) adult patient (≥ 18 years), (ii) having started treatment with trabectedin at a dose of 1.5 mg/m^2^ (the approved schedule) before 31/12/2021, regardless of the duration of treatment, (iii) patient previously treated with anthracyclines, (iv) metastatic disease or locally advanced not amenable to curative intent surgery, (v) patient with leiomyosarcoma or liposarcoma, (vi) primary site in soft tissue or viscera (including uterus), and (vi) patient treated in one of five participating centers. The exclusion criteria were: (i) non‐evaluable or non‐measurable disease according to RECIST before starting trabectedin, and (ii) combination therapy with trabectedin (including concurrent radiotherapy).

### 2.3 Endpoints

Details of the computation of the RDI are provided as Supporting Information. We classified as low‐RDI an RDI < 80% [45].

The primary endpoint for the prognostic factor analyses was progression‐free survival (PFS) considering as events progression (according to RECIST 1.1) or death. Observations were censored if a second line treatment was started before the date of reported progression. In the absence of any event, observations were censored at the date of the last news.

We studied the overall survival (OS) as a secondary endpoint, considering death from any cause. Observations were censored at the date of the latest news for patients still alive.

### 2.4 Statistical Considerations

Patient and disease characteristics were presented overall and according to whether or not patients were included in the main analysis population, and according to the RDI over the first three cycles (< 80% vs. ≥ 80%). Distribution of continuous variables was described by the median and the range, and by the mean and the standard deviation (mean ± SD). Data were compared between subgroups using Chi‐square or Fisher exact tests for categorical variables, and Wilcoxon–Mann–Whitney test for continuous variables.

PFS and OS probabilities were estimated usingthe Kaplan‐Meier method, overall and according to RDI (< 80% vs. ≥ 80%).

The association between RDI and PFS/OS was estimated by Hazard Ratio (HR) and tested in Cox models. The univariable analysis was completed by a multivariable analysis controlling for possible confounders.

The study population consists of all eligible patients who have started trabectedin. The main analysis was restricted to patients who have received at least 3 cycles of trabectedin with no progression before the start of the third cycle, considering the RDI computed over the first 3 cycles. We performed a first sensitivity analysis on patients who received at least 2 cycles of trabectedin with no progression before the start of the second cycle, considering the RDI computed over the first 2 cycles. For these analyses, the time interval for survival estimates was computed from the first trabectedin cycle.

We performed a second sensitivity analysis to control for a possible guarantee‐time bias. Indeed, patients with low RDI over the first 3 cycles could be patients with prolonged time intervals between cycles; their survival times would be mechanically longer than those of patients with a higher RDI. In this second sensitivity analysis, we computed the survival times (PFS and OS) from the end of the third cycle, defined by the date of the fourth cycle if administered, otherwise the start of the third cycle +21 days. If an event occurred before that date, we considered the day before the event date as the starting date to compute the survival times.

We performed a third sensitivity analysis considering a threshold of 90% to define low versus high RDI (< 90% vs. ≥ 90%). Lastly, we explored the dose effect relationship between RDI and PFS considering the quartiles of the observed distribution of RDI in the study population.

The software used is Stata version 17.0 (StataCorp. 2017. Stata Statistical Software: Release 15. College Station, TX: StataCorp LLC).

### 2.5 Regulatory Considerations

The data were collected from hospital files without any interaction with patients for research. The Commission for Clinical Studies of the Oscar Lambret Center has approved the study (CEC‐2022‐018) and has confirmed that no ethical approval by an independent ethics committee was required for the study. The study complies with the Reference Methodology MR004 of the National Commission on Informatics and Freedoms (CNIL “Commission Nationale Informatique et Liberté”). We have checked that no patient objected to the use of their medical data for research purposes.

## 3 Results

We have collected data from 332 patients having started trabectedin between 09/1999 and 12/2021 (Figure S1; Table S1). Among these 332 patients, 244 (73.5%) have received at least 3 cycles with no progression before the third cycle (main analysis), and 304 (91.6%) have received at least 2 cycles (first sensitivity analysis). The patient and tumor characteristics are summarized in Table 1. More than 2/3 of patients have been enrolled in 2 high‐activity centers (Centre Oscar Lambret and Centre Léon Bérard). Patients who have not been included in the main analysis, that is, those who have not received 3 trabectedin cures for any cause, as well as those who progressed before the third cycle, were characterized, as expected, by poor general condition (high ECOG performance status, low albumin levels, elevation of alkaline phosphatase …) and aggressiveness of sarcoma (short interval between initial diagnosis and onset of trabectedin).

**TABLE 1:** Patients and tumors characteristics at the time of first trabectedin cycle.

Characteristics	RDI ≥ 80% in the main analysis (*N* = 138)		RDI < 80% in the main analysis (*N* = 106)		Study population *N* = 244		*p* ^a^
	*N*	%	*N*	%	*N*	%	
Center							0.03
Center Eugène Marquis, Rennes	11	8.0%	10	9.4%	21	8.6%	
Center Léon Bérard, Lyon	49	35.5%	38	35.8%	87	35.7%	
Center Oscar Lambret, Lille	38	27.5%	44	41.5%	82	33.6%	
ICANS, Strasbourg	7	5.1%	3	2.8%	10	4.1%	
Institut de Cancérologie de l'Ouest, Nantes	33	23.9%	11	10.4%	44	18.0%	
Sex							0.80
Male	46	33.3%	37	34.9%	83	34.0%	
Female	92	66.7%	69	65.1%	161	66.0%	
Age at 1st cycle							0.86
Median – (Range)	59.4	(26.0; 81.1)	59.4	(31.9; 76.9)	59.4	(26.0; 81.1)	
Mean – SD	58.2	11.6	58.1	10.9	58.1	11.3	
ECOG performance status score	*N* = 124		*N* = 97		*N* = 221		0.99^b^
0	40	32.3%	31	32.0%	71	32.1%	
1	72	58.1%	56	57.7%	128	57.9%	
2	12	9.7%	9	9.3%	21	9.5%	
3	0	0.0%	1	1.0%	1	0.5%	
Body Mass Index (kg/m^2^)							0.69
Underweight: < 18.5	9	6.5%	4	3.8%	13	5.3%	
Normal: 18.5–25	68	49.3%	52	49.1%	120	49.2%	
Overweight: 25–30	35	25.4%	32	30.2%	67	27.5%	
Obesity: > 30	26	18.8%	18	17.0%	44	18.0%	
Albuminemia	*N* = 71		*N* = 53		*N* = 124		0.02^c^
Standard	50	70.4%	47	88.7%	97	78.2%	
Grade 1 hypoalbuminemia	18	25.4%	4	7.5%	22	17.7%	
Grade 2 hypoalbuminemia	2	2.8%	2	3.8%	4	3.2%	
Grade 3 hypoalbuminemia	1	1.4%	0	0.0%	1	0.8%	
Alkaline phosphatase	*N* = 114		*N* = 89		*N* = 203		0.82^c^
Standard	100	87.7%	79	88.8%	179	88.2%	
Grade 1 increase	14	12.3%	9	10.1%	23	11.3%	
Grade 2 increase	0	0.0%	1	1.1%	1	0.5%	
Grade 3 increase					0	0.0%	
Creatinine	*N* = 118		*N* = 89		*N* = 207		0.41^c^
Standard	93	78.8%	65	73.0%	158	76.3%	
Grade 1 increase	24	20.3%	24	27.0%	48	23.2%	
Grade 2 increase	1	0.8%	0	0.0%	1	0.5%	
Primary Site							0.52
Soft tissue	92	66.7%	78	73.6%	170	69.7%	
Uterus	44	31.9%	27	25.5%	71	29.1%	
Missing	2	1.4%	1	0.9%	3	1.2%	
Histological subtypes							0.52
Leiomyosarcoma	82	59.4%	69	65.1%	151	61.9%	
Myxoid and round cell liposarcoma	17	12.3%	14	13.2%	31	12.7%	
Other liposarcoma	39	28.3%	23	21.7%	62	25.4%	
FNCLCC grading System							0.74
Grade 1	15	10.9%	10	9.4%	25	10.2%	
Grade 2	45	32.6%	32	30.2%	77	31.6%	
Grade 3	39	28.3%	37	34.9%	76	31.1%	
Missing or not applicable	39	28.3%	27	25.5%	66	27.0%	
Number of prior lines	*N* = 137		*N* = 105		*N* = 242		0.90
Median–(Range)	1	(0; 6)	1	(0; 4)	1.0	(0.0; 6.0)	
Mean–SD	1.5	0.9	1.5	1.0	1.5	0.9	
Prior Treatments							
Doxorubicine	133	96.4%	99	93.4%	232	95.1%	0.29
Ifosfamide	66/137	48.2%	40	37.7%	106/243	43.6%	0.10
Dacarbazine	21/137	15.3%	25	23.6%	46/243	18.9%	0.10
Pazopanib	6/137	4.4%	12	11.3%	18/243	7.4%	0.04
Other drugs	66	47.8%	51	48.1%	117	48.0%	0.97
Time interval between initial diagnosis and 1st cycle of trabectedin (years)							0.49
Median–(Range)	2.1	(0.2; 17.2)	2.6	(0.1; 21.6)	2.3	(0.1; 21.6)	
Mean–SD	3.6	3.7	3.8	3.9	3.7	3.8	
Metastasis	*N* = 138		*N* = 106		*N* = 244		0.25
No	10	7.2%	4	3.8%	14	5.7%	
Yes	128	92.8%	102	96.2%	230	94.3%	
Metastatic sites	*N* = 136		*N* = 106		*N* = 242		0.18
Number of metastatic sites							
Median–(Range)	2	(0; 6)	2	(0; 7)	2	(0; 7)	
Mean–SD	1.9	1.2	2.2	1.3	2.0	1.3	
Liver metastasis	37	26.8%	38	35.8%	75	30.7%	0.13
Lung metastasis	71	51.4%	61	57.5%	132	54.1%	0.34
Peritoneal metastasis	53	38.4%	33	31.1%	86	35.2%	0.24

*Note:* Rounding can lead to a sum of the percentages in columns greater than 100%. Abbreviations: ECOG, eastern cooperative oncology group; MD, missing data.

^a^ For qualitative variables, Chi‐square test performed when the application conditions (theoretical numbers ≥ 5) were met, otherwise the exact Fisher test was applied. For quantitative variables, comparison by Wilcoxon test.

^b^ Testing the distribution in 3 categories by grouping ECOG score equal to 2 and 3 versus ECOG equal to 1 versus ECOG equal to 0.

^c^ Testing the distribution in 2 categories normal versus abnormal.

**FIGURE 1:**
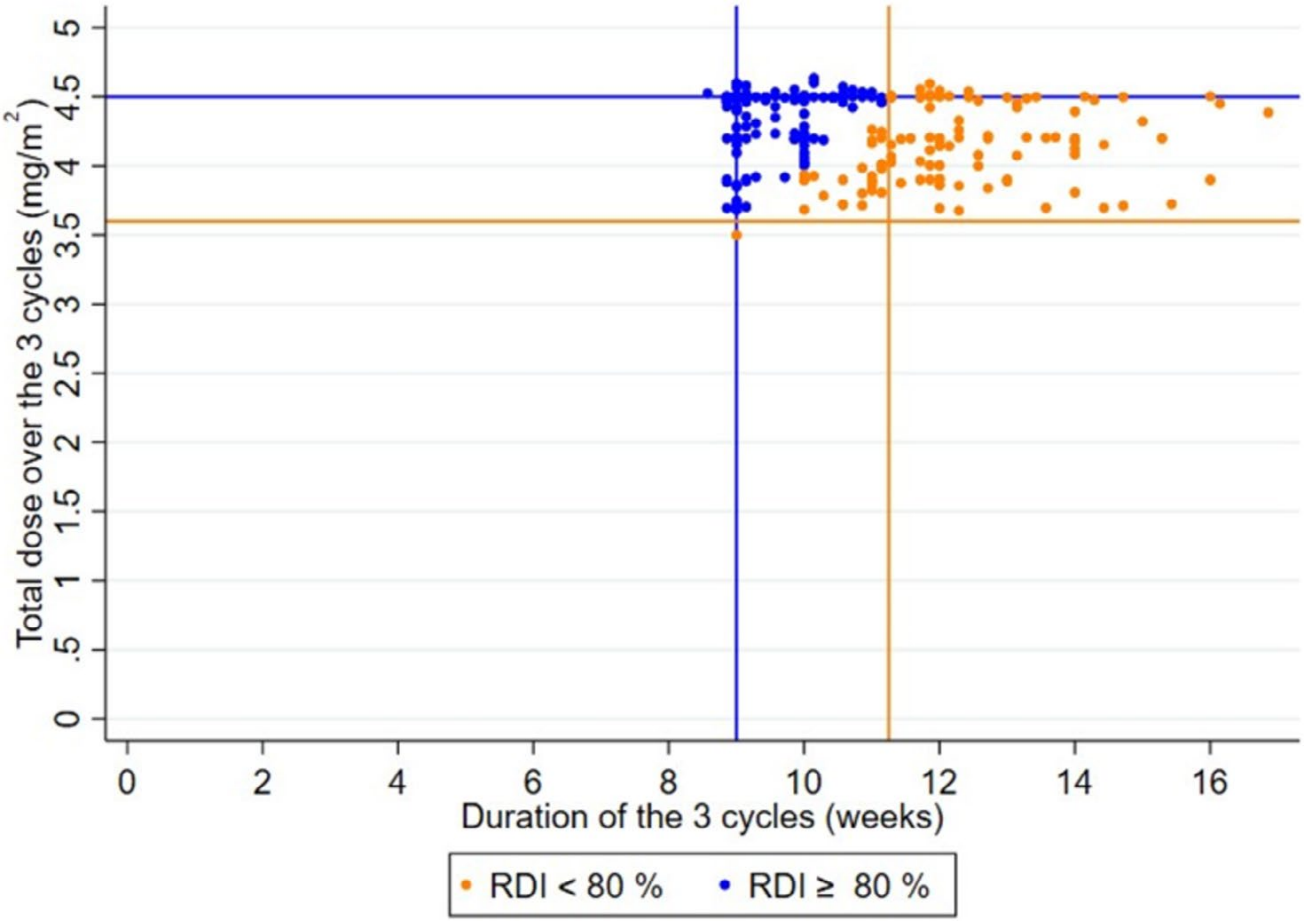
Scatter plot of the cumulative dose received based on duration of treatment for the first 3 treatment cycles for patients who received at least 3 cycles of trabectedin (*N* = 244). In this scatter plot, each point represents one patient. The blue points represent patients with a computed RDI ≥ 80%, The orange points represent patients with a computed RDI < 80%. The vertical lines represent two values for the duration of 3 cycles. In blue, the expected duration of 9 cycles. In orange, a prolonged duration of 11.25 weeks (which could lead to an RDI < 80%) due to delayed courses. The horizontal lines represent two values for the cumulative trabectedin dose over 3 cycles. In blue, the expected dose of 4.5 mg/m^2^. In orange, a reduced dose of 3.6 mg/m^2^ (which could lead to an RDI < 80%). Overall, 106 patients (43.4%) had an RDI < 80%. For 87 patients (82%), the main factor leading to a reduced RDI was the larger time intervals between cycles, leading to a prolonged duration > 11.25 weeks, whereas the cumulative dose was maintained > 3.6 mg/m^2^. Only 1 patient (< 1%) had major dose reductions with a cumulative dose < 3.6 mg/m^2^, but no delayed courses. For the 18 patients (17%), the reduced RDI was due to a combination of slightly larger time intervals between cycles and slightly reduced doses.

Overall, the median follow‐up was 101.0 months (interquartile range, 63.2 to 143.3 months). The median PFS of the entire cohort of 332 patients was 4.7 months (95% Confidence Interval, 4.1–5.7). The median overall survival of the entire cohort was 14.5 months (12.6–16.1). Among these 244 patients enrolled in the main analysis, the number of trabectedin cycles was 3 to 47 (median = 6, range 3–47, mea*n* = 8.0 ± 5.7). The median duration of trabectedin treatment was 4.7 months (range 1.3–53.7) and the mean duration of treatment was 6.1 months ±5.6. All patients have stopped trabectedin treatment at the time of the analysis. The reasons for trabectedin discontinuation were as follows: disease progression (*n* = 165, 67.6%), drug holiday (*n* = 64; 26.2%), toxicity (*n* = 8, 3.3%), lost to follow‐up (*n* = 2, 0.8%), death (*n* = 1, 0.4%), intercurrent cardiac event (*n* = 1, 0.4%) and unknown (*n* = 3, 1.2%). The expected cumulative trabectedin dose over the first 3 cycles is 4.5 mg/m^2^. As illustrated by Figure 1, we observed a larger variation in terms of the time interval between cycles than in the dose administered. The median cumulative administered trabectedin dose over the first 3 cycles was 4.4 mg/m^2^ (range 3.5–4.6), and the mean dose was 4.3 mg/m^2^ ± 0.3. The expected duration of treatment over the first 3 cycles is 9 weeks. The observed median duration of treatment over the first 3 cycles was 10.3 weeks (range, 8.6–16.9), and the mean duration was 10.7 months ±1.8. As a consequence, the median RDI was 82.7% (range, 48.3–105.6) and the mean RDI was 81.4% ± 14.0. Overall, 106 patients (43.4%) had an RDI < 80%, mainly due to a larger time interval between cycles (87/106 patients, 82%) (Figure 1).

Two patient or tumor characteristics were associated with RDI < 80%: to be treated in a center with a high volume of activity (*p* = 0.03) and pre‐exposure to pazopanib (*p* = 0.04). Furthermore, the observed toxicity during the 1st cycle was associated with RDI < 80% (Table 2). The occurrence of grade 3 or higher toxicity was significantly associated with an RDI < 80% (*p* < 0.001). This was especially marked for severe neutropenia. Among the 204 patients informative for neutropenia, 77 experienced grade ≥ 3 neutropenia (37.7%) after the first cycle, and 56 of these 77 patients (72.7%) had RDI < 80%, contrasting with 35 of the 127 patients (27.6%) who had no neutropenia or only grade 1–2 neutropenia (*p* < 0.001). On the contrary, transaminase increase, which was also frequently observed after the first cycle (27.7% and 13.3% of informative patients, for ALAT and ASAT, respectively) was not significantly associated with subsequent RDI reduction (*p* = 0.17 and 0.76, respectively).

**TABLE 2:** Probability of reduced RDI over the 3 first cycles according to observed toxicity during the 1st cycle of trabectedin for patients who received at least 3 cycles of Trabectedin.

Nature of toxicity	No toxicity *n* (RDI < 0.8)/*N* ^a^ (%)	Grade 1 or 2 *n* (RDI < 0.8)/*N* ^b^ (%)	Grade ≥ 3 *n* (RDI < 0.8)/*N* ^c^ (%)	*p* ^d^
Any type	5/12 (41.6%)	31/115 (26.9%)	70/116 (60.3%)	< 0.001
Nausea	66/158 (41.7%)	33/78 (42.3%)	3/3 (100%)	
Vomiting	86/209 (41.1%)	13/27 (48.1%)	2/2 (100%)	
Extravasation	103/238 (43.2%)	0/2 (0%)		
ALAT Increase	22/59 (37.2%)	51/104 (49.0%)	12/25 (48.0%)	0.76
ALAT Increase	15/42 (35.7%)	43/94 (45.7%)	28/52 (53.8%)	0.17
Alkaline Phosphatase Increase	59/127 (46.4%)	22/46 (47.8%)	0/1 (0%)	
GGT Increase	26/79 (32.9%)	41/78 (52.5%)	13/22 (59.0%)	0.15
Bilirubin Increase	86/189 (45.5%)	2/5 (40.0%)		
Asthenia	67/157 (42.6%)	27/66 (40.9%)	3/8 (37.5%)	
Myalgia	99/229 (43.2%)	2/8 (25.0%)	1/1 (100%)	
Increase in CPK	47/95 (49.4%)	2/4 (50.0%)		
Renal failure	75/175 (42.8%)	12/27 (44.4%)		
Neutropenia	19/86 (22.0%)	16/41 (39.0%)	56/77 (72.7%)	< 0.001
Febrile neutropenia	94/218 (43.1%)		2/2 (100%)	
Thrombocytopenia	64/162 (39.5%)	21/33 (63.6%)	3/4 (75.0%)	
Anemia	30/63 (47.6%)	59/136 (43.3%)	2/4 (50.0%)	
Anorexia	86/212 (40.5%)	12/21 (57.1%)	1/1 (100%)	
Peripheral oedema	98/232 (42.2%)	1/3 (33.3%)		
Diarrhea	98/227 (43.1%)	3/9 (33.3%)	1/2 (50.0%)	
Constipation	96/219 (43.8%)	6/18 (33.3%)	0/1 (0%)	
Rash	102/237 (43.0%)	0/1 (0%)		

*Note:* When the numbers of patients are too small or equal to zero in certain groups, the *p*‐values are not given.

^a^ *N* is the total number of patients who did not experience toxicity during the first cycle and *n* is the number of patients with an RDI < 80% over the 3 first cycles among these *N* patients.

^b^ *N* is the total number of patients with a maximum grade of 1 or 2 during the first cycle and *n* is the number of patients with an RDI < 80% over the 3 first cycles among these *N* patients.

^c^ *N* is the total number of patients with a maximum grade ≥ 3 during the first cycle, and *n* is the number of patients with an RDI < 80% over the 3 first cycles among these *N* patients.

^d^ *p*‐value associated with Chi‐square tests comparing the distribution of severe toxicity versus absent/grade 1 or 2 toxicity in patients with RDI < 80% versus RDI ≥ 80%.

**FIGURE 2:**
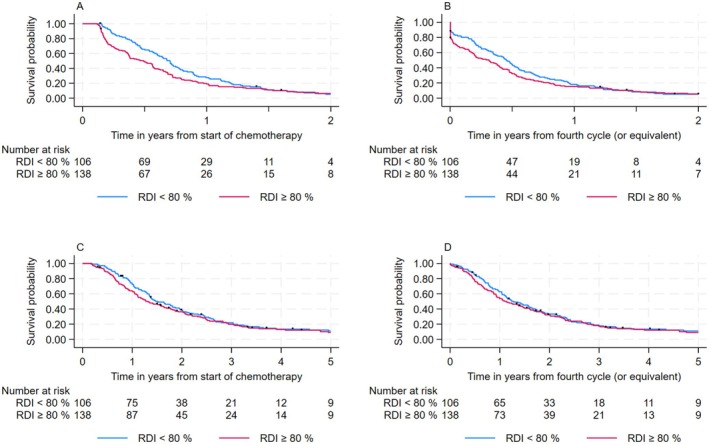
(A) Progression‐free survival from the start of chemotherapy according to relative dose intensity over the first 3 cycles, in patients who received at least 3 cycles of trabectedin. (B) Progression free survival from the fourth cycle (or equivalent) according to relative dose intensity over the 3 first cycles, in patients who received at least 3 cycles of trabectedin (second sensitivity analysis). (C) Overall survival from start of chemotherapy according to relative dose intensity over the 3 first cycles, in patients who received at least 3 cycles of trabectedin. (D) Overall survival from the fourth cycle (or equivalent) according to relative dose intensity over the 3 first cycles, in patients who received at least 3 cycles of trabectedin from the fourth cycle (second sensitivity analysis).

The median PFS of the selected 244 patients who had received at least 3 cycles was 6.9 months from the start of trabectedin (95% CI, 5.9–7.8). As illustrated by Figure 2A and detailed in Table S2, PFS did not significantly differ according to RDI: the hazard ratio associated with an RDI < 0.80 was 0.79 (0.61–1.03) with a *p*‐value of 0.08; median PFS = 5.9 months (4.4–6.8) when RDI ≥ 80% vs. 8.4 months (7.0–9.3) when RDI < 80%.

Among the 244 patients included in the main analysis who had received at least 3 trabectedin cycles, the median overall survival was 17.0 months (15.1–20.0). As illustrated by Figure 2C and detailed in Table S2, we observed no significant difference in terms of OS according to RDI: the HR associated with RDI < 0.80 was 0.92 (0.70–1.20) with a *p*‐value of 0.53; median OS = 15.8 months (13.2–19.7) when RDI ≥ 80% vs. 18.2 months (15.6–23.4) when RDI < 80%.

Lastly, we have carried out adjusted analyses taking into account prior exposure to pazopanib and high‐activity centers that are both associated with an RDI < 80% (Table 1). In the multivariate analysis of PFS, HR associated with RDI < 0.80 was 0.77 (0.60–1.00; *p* = 0.053), HR associated with high‐activity centers was 1.17 (0.88–1.55; *p* = 0.28), and HR associated with prior exposure to pazopanib was 0.98 (0.59–1.64; *p* = 0.95). In the same way for the OS, HR associated with RDI < 0.80 was 0.90 (0.68–1.19; *p* = 0.46), HR associated with high‐activity centers was 1.21 (0.89–1.66; *p* = 0.21), and HR associated with prior exposure to pazopanib was 0.81 (0.48–1.38; *p* = 0.44).

We conducted a first sensitivity analysis of the 304 patients who received at least 2 trabectedin cycles. The median PFS was 5.3 months (4.5–6.6). In this sensitivity analysis, we observed a significantly better PFS in patients with low RDI: the HR associated with RDI < 0.80 was 0.75 (0.59–0.95, *p* = 0.02); the median PFS was 7.8 months (95% CI, 5.6–8.7) in the RDI < 80% group and 4.4 months (2.8–5.5) in the RDI ≥ 80% group. Considering this data set of 304 patients, the overall survival at 1 year was 61.9% (56.2–67.1). There was no significant difference in overall survival in patients with low RDI (RDI < 80%) compared to other patients: the HR associated with RDI < 0.80 was 0.86 (0.68–1.11; *p* = 0.25); the median overall survival was 17.3 months (15.6–22.4) in patients with RDI < 80% and 14.1 months (12.4–15.5) in patients with RDI ≥ 80%.

When considering the second sensitivity analysis (Table S2), carried out to better control a possible guarantee‐time bias, the difference of PFS between patients with high versus low RDI was smaller than in the primary analysis: HR_(< 80%/≥ 80%)_ = 0.87 (0.67–1.12), *p* = 0.27 (Figure 2B). The same observation applies to the OS analysis: HR_(< 80%/≥ 80%)_ = 0.95 (0.72–1.24), *p* = 0.69 (Figure 2D).

As illustrated by Figure S2, we observed a slightly larger PFS difference between the two groups when we used the threshold of RDI < 90% to define a low RDI: the HR associated with RDI < 90% was 0.79 (0.60–1.04) with a *p*‐value of 0.09; median PFS = 4.6 months (3.4–6.7) when RDI ≥ 90% vs. 7.8 months (6.8–8.5) when RDI < 90%.

This is in accordance with a trend for a better PFS with reduced RDI as illustrated by the analysis considering the quartiles of the distribution (*p* = 0.06; Figure S3).

## 4 Discussion

This retrospective study conducted in five labeled sarcomas centers in France does not support a link between high RDI and better PFS or OS for advanced L‐sarcomas patients. These results are stable, whether we calculated the RDI over the first 2 or 3 cycles, or even adjusted for factors associated with a decrease in RDI (prior exposure to pazopanib or management in a high‐volume center). The most frequent toxicities were hematological and liver toxicities, well known with trabectedin and similar to the pivotal phase III study of Demetri [25]. An RDI < 80% was more frequently observed in patients who experienced grade 3 neutropenia during the first cycle.

Our study is the first to evaluate the association between trabectedin RDI and outcomes in patients treated for L‐sarcoma. The data from our cohort were in line with those found in the literature. Demographic characteristics were as expected for L‐sarcoma patients: median age of 60 years old [9, 25, 46, 47], a sex ratio M/F < 1 consistent with a high number of uterine LMS [44, 48], a Performance Status ≤ 1 in more than 75% of the patients [46, 47, 49], and the majority of high‐grade sarcomas [9, 46]. Outcomes were also comparable to literature data. With a median follow‐up time of 101.0 months, we observed a median (overall) PFS of 4.7 months and a median OS of 14.5 months from the first cycle of trabectedin. In particular, we should mention the pivotal phase III study by Demetri et al., which found a median PFS of 4.2 months in the trabectedin arm, and the study by Le Cesne et al., which found a median PFS of 5.1 months in the subgroup of patients with L‐sarcomas [9, 25]. The retrospective multicenter study by the Italian Sarcoma Group published in 2021 found a median PFS of 5.1 months (it should be noted, however, that this study also evaluated other anatomopathological subtypes, with the L‐sarcoma subgroup accounting for 68% with a PFS between 8 and 9 months) [47]. Hematological and liver toxicities found in our study were close to those from the Demetri et al. study, with 37% of patients experiencing grade ≥ 3 neutropenia and 26%/15% experiencing ALAT/ASAT increase. Other clinical toxicities such as nausea, fatigue, diarrhea, and constipation were less frequent in our study, probably because of the retrospective nature of our study.

RDI is rarely reported in literature. The median RDI of 82.7% over the first 3 cycles found in our study was between data reported in the phase III trials (85%–90%) and the data reported in the phase IV study of Buonadonna et al. and the retrospective study of Saito et al. (75%) [9, 29, 45, 50]. It should be noted that in our study, RDI was calculated only over the first three cycles. In our study, RDI < 80% (over the 3 first cycles) was seen in 43% of patients and 35% in the Le Cesne et al. study. In our study, the main mechanism of RDI reduction corresponded to an increase in the duration between two cycles of treatment, which was also stressed by Grünwald et al. [46].

The strength of the present study was its multicenter nature, based on real‐life data, with a relatively large sample size. It enables us to answer a bedside everyday practice question, which could have a direct impact on patients' health‐related quality of life. However, it also has several limitations. Inaccuracies in patient records and missing data inherent (e.g., precise description of toxicities) in the retrospective nature of the data collection are necessarily sources of measurement bias. It should be noted that it was decided a priori that the RDI would be calculated up to the first scan evaluation (after 2 or 3 cycles), to limit possible guarantee‐time bias when considering further treatment. However, in real‐life practice, the date of the assessment scans is subject to considerable inter‐individual variability, making it challenging to estimate both PFS and RDI. Another issue in the evaluation of the association between RDI and survival outcomes is that patients with low RDI over the first cycles were mostly patients with prolonged time intervals between cycles (Figure 1); their progression‐free survival was mechanically longer than that of patients with a higher RDI, contributing to a guarantee‐time bias when the survival curves were estimated from the first trabectedin cycle; we performed another sensitivity analysis to control this bias. Quality of life data were not available in our study, as well as the concomitant treatments taken by patients. For a significant number of patients, the initial prescription did not comply with the recommended dose, reducing retrospective recruitment in our study. In particular, no patients could be recruited at the Centre Leon Berard in Lyon after 2016, as Trabectedin has been prescribed at an initial dose of 1.2 mg/m^2^ since that date. We have used the threshold of 80% to define lower RDI; this threshold was chosen based on numerous prior studies assessing RDI of trabectedin in soft tissue sarcoma management [9, 29, 45, 50]. This threshold is debatable. However, results regarding the association between RDI and PFS appear consistent when considering a threshold of 90% to define a low RDI. Actually, when considering the quartiles of the distribution of RDI, we observed a trend for a better PFS with reduced RDI, suggesting a continuum (Figure S3).

At the end, the current approved dose could be discussed. Indeed, 1.5 mg/m^2^/3w was determined from the phase I study of Taama et al. [28]. With that dosage, most of the hematological toxicities were reversible in one week, rarely associated with complications. Nevertheless, in this phase I study, 48% of cycles were delayed, 35% of which were caused by hematologic toxicity, principally neutropenia.

Since an RDI of 80% for a standard dose of 1.5 mg/m^2^ corresponds in fact to a dose of 1.2 mg/m^2^/3 weeks, the question arises of reducing this standard treatment dose to 1.2 mg/m^2^/3 weeks, in order to improve tolerance to treatment in the context of palliative chemotherapy. In the study by Taama et al., there was no severe hematological toxicity at this dose. Following the example of the Leon Berard Center already mentioned, the Gustave Roussy Institute in Paris has been reducing the initial treatment dose to 1.2 mg/m^2^ on an empirical basis for several years. It should be noted that 1.2 mg/m^2^/3 weeks was the dose recommended in a Japanese phase I study [51]. The retrospective study by Palmerini et al. also found no significant difference in PFS if the starting dose was 1.3 mg/m^2^, subject to the low power of this test, which was not foreseen a priori [47]. In the study by Saito et al., the use of Pegfilgrastim was not associated with a higher RDI, neither a higher number of cycles [45]. Lastly, Ön et al. also showed in a retrospective study of 98 cases that dose reductions were not associated with poorer OS [52].

Ten years after its approval, the optimal schedule of trabectedin administration remains an open question in the management of L‐sarcoma. Trabectedin is one of the few drugs approved in cases of failure, intolerance, or contraindication to doxorubicin. However, recently, the doxorubicin‐trabectedin combination compared to doxorubicin demonstrated in a phase III study its ability to very markedly improve the overall survival of patients with advanced L‐sarcoma [53]. This improvement in survival is an unprecedented breakthrough. In 30 years of clinical research, no treatment has provided such benefit for sarcoma patients. In this phase III study, trabectedin was administered with a different schedule of 1.1 mg/m^2^ over 3 h every 3 weeks, in combination with doxorubicin and as maintenance therapy. We must continue to work to optimize the schedule of administration of this major drug.

Our study does not demonstrate a link between high trabectedin RDI and patient outcome. In a context of palliative care, where the question of limiting adverse effects is of crucial importance, it seems important to us to continue these post‐approval clinical explorations in order to find for each clinical situation the best dose compromise between efficacy and toxicity. Second‐ and third‐line trabectedin monotherapy aims to both slow the progression of sarcoma and maintain quality of life. Dose adaptation in the routine treatment of palliative chemotherapy enables better tolerance and efficacy of treatments. Of course, this is a specific situation; our conclusions do not apply to curative situations such as the neoadjuvant treatment of Ewing sarcomas [54].

## Author Contributions

**Stephen Poitureau:** conceptualization; investigation; writing – original draft; writing – review and editing. **Marie‐Cécile Le Deley:** writing – review and editing; conceptualization; methodology; software; data curation; validation; formal analysis. **Mehdi Brahmi:** writing – review and editing; investigation. emmanuelle bompas: writing – review and editing; investigation. **Jean‐Emmanuel Kurtz:** writing – review and editing; investigation. **Simon Nannini:** writing – review and editing; investigation. **Christophe Perrin:** writing – review and editing; investigation. loïc lebellec: writing – review and editing; investigation. **Thomas Ryckewaert:** writing – review and editing; investigation. **Redha Ould‐Ammar:** writing – review and editing; software; data curation; formal analysis. **Clémence Leguillette:** writing – review and editing; methodology; data curation; formal analysis. **Jean‐Yves Blay:** writing – review and editing; investigation. **Nicolas Penel:** writing – review and editing; conceptualization; investigation; validation; supervision; funding acquisition; visualization; project administration; resources; writing – original draft.

## Ethics Statement

Ethical approval for this study was not required. The study complies with the reference methodology MR004 adopted by the French Data Protection Authority (CNIL). None of the patients had objected to the use of their clinical data for research purposes.

## Conflicts of Interest

The authors declare no conflicts of interest.

## Supporting information


**Data S1:** cam471131‐sup‐0001‐DataS1.docx.

## Data Availability

The data that support the findings of this study are available on request from the corresponding author. The data are not publicly available due to privacy or ethical restrictions.
