# Supplementary material for: Relative Dose Intensity of Trabectedin and Outcome of Advanced L‐Sarcomas
Source: Cancer Med. 2025 Aug 19;14(16):e71131. doi: 10.1002/cam4.71131 (PMC12361930; doi:10.1002/cam4.71131)
Supplement: Supplementary file 1 — Data S1: cam471131‐sup‐0001‐DataS1.docx. [file CAM4-14-e71131-s001.docx]

SUPPLEMENTARY DATA

## Relative-dose intensity computation

Relative dose-intensity was calculated using the following formula:

$${RDI}_{k}= \frac{\frac{\sum_{i=1}^{i=n} D_{i}}{t_{k}}}{0.5}$$

$D_{i}$ : Dose per body surface area (BSA) received at cycle i, considering a body surface area of 2 for patients with actual BSA greater than 2. The Body Surface Area was calculated using the Dubois formula (Body Surface Area = 0.007184*Height (cm)^0.725^ * weight (kg)^0.425^.

$t_{k}$ : Time-interval between the first cycle until the cycle k+1 if administered, otherwise the time-interval between the first cycle and the cycle k + 21 days.

Since the theoretical dose is 1.5 mg/m² every 3 weeks, the dose intensity observed on the first k cycles ($\frac{\sum_{i=1}^{i=n} D_{i}}{t_{k}}$ expressed in mg/m²/week) is divided by 0.5 mg/m²/week to obtain the Relative Dose Intensity.

## Figure S1 – Flow Chart


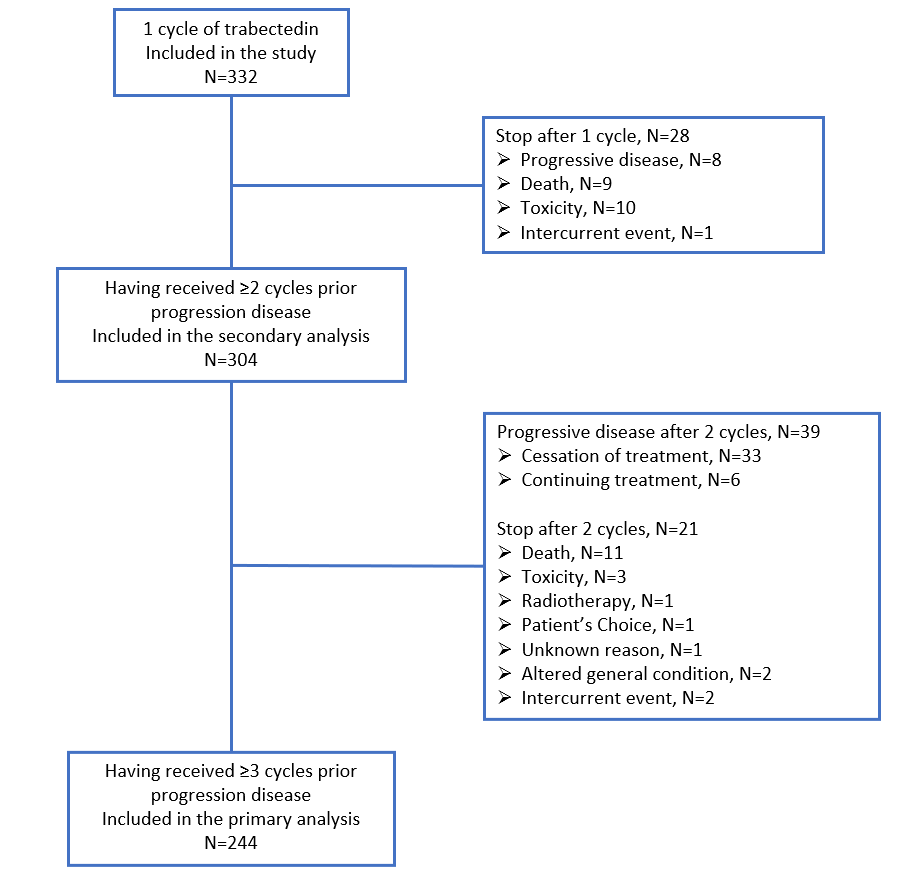


## Figure S2 – PFS in patients with RDI<90% versus patients with RDI≥90%
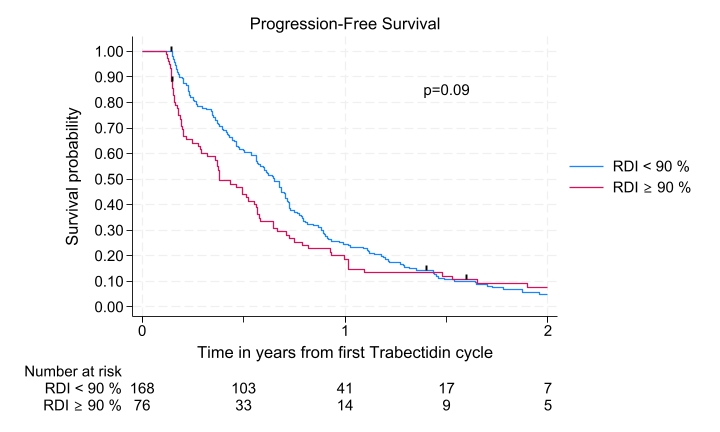


## Figure S3 – PFS according to quartile of RDI over the first 3 cycles


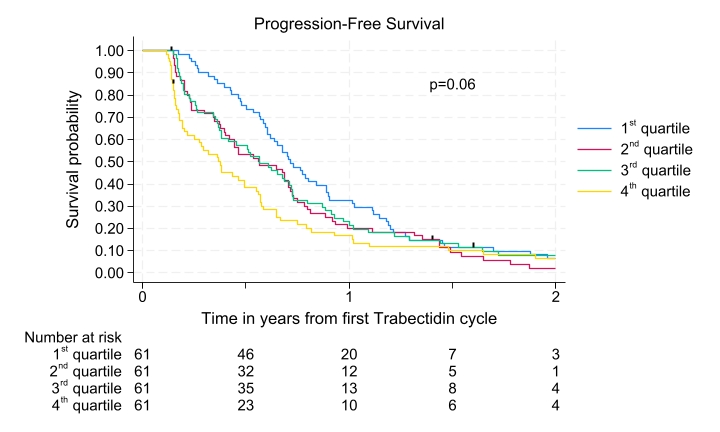


The 1^st^ quartile corresponds to lower RDI, with observed values [0.48-0.703]; the 2^nd^ quartile ]0.703-0.826], the 3^rd^ quartile ]0.826-0.930], whilst the 4^th^ quartile corresponds to higher RDI values ]0.930-1.06].

## Table S1 – Patients and tumours characteristics at the time of first trabectedin cycle patients included in the main analysis versus not included in the main analysis

| Characteristics | Patients included in the main analysis N=244 | | Patients not included in the main analysis N=88 | | Overall population   N=332 | | p-value^(1)^ |
| --- | --- | --- | --- | --- | --- | --- | --- |
|  | N | % | N | **%** | N | % |  |
| Centre |  |  |  |  |  |  | 0.15 |
| Centre Eugène Marquis, Rennes | 21 | 8.6% | 9 | 10.2% | 30 | 9.0% |  |
| Centre Léon Bérard, Lyon | 87 | 35.7% | 43 | 48.9% | 130 | 39.2% |  |
| Centre Oscar Lambret, Lille | 82 | 33.6% | 25 | 28.4% | 107 | 32.2% |  |
| ICANS, Strasbourg | 10 | 4.1% | 1 | 1.1% | 11 | 3.3% |  |
| Institut de Cancérologie de l’Ouest, Nantes | 44 | 18.0% | 10 | 11.4% | 54 | 16.3% |  |
| Sex |  |  |  |  |  |  | 0.69 |
| Male | 83 | 34.0% | 32 | 36.4% | 115 | 34.6% |  |
| Female | 161 | 66.0% | 56 | 63.6% | 217 | 65.4% |  |
| Age at 1st cycle |  |  |  |  |  |  | 0.08 |
| Median - (Range) | 59.4 | (26.0 ; 81.1) | 61.3 | (31.7 ; 79.5) | 60.0 | (26.0 ; 81.1) |  |
| Mean – SD | 58.1 | 11.3 | 60.8 | 12.1 | 58.8 | 11.5 |  |
| ECOG performance status score | N=221 |  | N=73 |  | N=294 |  | <0.001^(2)^ |
| 0 | 71 | 32.1% | 13 | 17.8% | 84 | 28.6% |  |
| 1 | 128 | 57.9% | 39 | 53.4% | 167 | 56.8% |  |
| 2 | 21 | 9.5% | 19 | 26.0% | 40 | 13.6% |  |
| 3 | 1 | 0.5% | 2 | 2.7% | 3 | 1.0% |  |
| Body Mass Index (kg/m²) |  |  |  |  |  |  | 0.60 |
| Underweight: <18.5 | 13 | 5.3% | 6 | 6.8% | 19 | 5.7% |  |
| Normal: 18.5 – 25 | 120 | 49.2% | 48 | 54.5% | 168 | 50.6% |  |
| Overweight: 25 – 30 | 67 | 27.5% | 23 | 26.1% | 90 | 27.1% |  |
| Obesity: >30 | 44 | 18.0% | 11 | 12.5% | 55 | 16.6% |  |
| Albuminemia | N=124 |  | N=53 |  | N=177 |  | <0.001^(3)^ |
| Standard | 97 | 78.2% | 26 | 49.1% | 123 | 69.5% |  |
| Grade 1 hypoalbuminemia | 22 | 17.7% | 15 | 28.3% | 37 | 20.9% |  |
| Grade 2 hypoalbuminemia | 4 | 3.2% | 11 | 20.8% | 15 | 8.5% |  |
| Grade 3 hypoalbuminemia | 1 | 0.8% | 1 | 1.9% | 2 | 1.1% |  |
| Alkaline phosphatase | N=203 |  | N=73 |  | N=276 |  | 0.04^(3)^ |
| Standard | 179 | 88.2% | 57 | 79.2% | 236 | 85.8% |  |
| Grade 1 increase | 23 | 11.3% | 13 | 18.1% | 36 | 13.1% |  |
| Grade 2 increase | 1 | 0.5% | 2 | 2.8% | 3 | 1.1% |  |
| Grade 3 increase | 0 | 0.0% | 1 | 1.4% | 1 | 0.4% |  |
| Creatinine | N=207 |  | N=77 |  | N=284 |  | 0.45^(3)^ |
| Standard | 158 | 76.3% | 62 | 80.5% | 220 | 77.5% |  |
| Grade 1 increase | 48 | 23.2% | 14 | 18.2% | 62 | 21.8% |  |
| Grade 2 increase | 1 | 0.5% | 1 | 1.3% | 2 | 0.7% |  |
| Primary Site |  |  |  |  |  |  | 0.91 |
| Soft tissue | 170 | 69.7% | 60 | 68.2% | 230 | 69.3% |  |
| Uterus | 71 | 29.1% | 27 | 30.7% | 98 | 29.5% |  |
| Missing | 3 | 1.2% | 1 | 1.1% | 4 | 1.2% |  |
| Histological subtypes |  |  |  |  |  |  | 0.38 |
| Leiomyosarcoma | 151 | 61.9% | 54 | 61.4% | 205 | 61.7% |  |
| Myxoid and round cell Liposarcoma | 31 | 12.7% | 7 | 8.0% | 38 | 11.4% |  |
| Other liposarcoma | 62 | 25.4% | 27 | 30.7% | 89 | 26.8% |  |
| FNCLCC grading System |  |  |  |  |  |  | 0.35 |
| Grade 1 | 25 | 10.2% | 7 | 8.0% | 32 | 9.6% |  |
| Grade 2 | 77 | 31.6% | 35 | 39.8% | 112 | 33.7% |  |
| Grade 3 | 76 | 31.1% | 29 | 33.0% | 105 | 31.6% |  |
| Missing or not applicable | 66 | 27.0% | 17 | 19.3% | 83 | 25.0% |  |
| Number of prior lines | N=242 |  | N=88 |  | N=330 |  | 0.22 |
| Median - (Range) | 1.0 | (0.0 ; 6.0) | 1.0 | (0.0 ; 3.0) | 1.0 | (0.0 ; 6.0) |  |
| Mean – SD | 1.5 | 0.9 | 1.4 | 0.8 | 1.5 | 0.9 |  |
| Prior Treatments |  |  |  |  |  |  |  |
| Doxorubicine | 232 | 95.1% | 87 | 98.9% | 319 | 96.1% | 0.20 |
| Ifosfamide | 106/243 | 43.6% | 31 | 35.2% | 137/331 | 41.4% | 0.17 |
| Dacarbazine | 46/243 | 18.9% | 11 | 12.5% | 57/331 | 17.2% | 0.17 |
| Pazopanib | 18/243 | 7.4% | 5/87 | 5.7% | 23/330 | 7.0% | 0.60 |
| other drugs | 117 | 48.0% | 37 | 42.0% | 154 | 46.4% | 0.34 |
| Time interval between initial diagnosis and 1st cycle of trabectedin (years) |  |  |  |  |  |  | 0.04 |
| Median - (Range) | 2.3 | (0.1 ; 21.6) | 1.7 | (0.3 ; 16.2) | 2.2 | (0.1 ; 21.6) |  |
| Mean – SD | 3.7 | 3.8 | 2.9 | 3.3 | 3.5 | 3.7 |  |
| Metastasis | N=244 |  | N=88 |  | N=332 |  | 0.99 |
| No | 14 | 5.7% | 5 | 5.7% | 19 | 5.7% |  |
| Yes | 230 | 94.3% | 83 | 94.3% | 313 | 94.3% |  |
| Metastatic sites | N=242 |  | N=88 |  | N=330 |  | 0.30 |
| Number of metastatic sites |  |  |  |  |  |  |  |
| Median - (Range) | 2 | (0 ; 7) | 2 | (0 ; 7) | 2 | (0 ; 7) |  |
| Mean - SD | 2.0 | 1.3 | 2.1 | 1.2 | 2.1 | 1.3 |  |
| Liver metastasis | 75 | 30.7% | 28 | 31.8% | 103 | 31.0% | 0.85 |
| Lung metastasis | 132 | 54.1% | 53 | 60.2% | 185 | 55.7% | 0.32 |
| Peritoneal metastasis | 86 | 35.2% | 34 | 38.6% | 120 | 36.1% | 0.57 |

MD=Missing Data; ECOG: Eastern Cooperative Oncology Group

Rounding can lead to a sum of the percentages in columns greater than 100 %.

1) For qualitative variables, Chi2 test performed when the application conditions (theoretical numbers ≥ 5) were met, otherwise the exact Fisher test was applied. For quantitative variables, comparison by Wilcoxon test.

2) Testing the distribution in 3 categories by grouping ECOG score equal to 2 and 3 versus ECOG equal to 1 versus ECOG equal to 0

3) Testing the distribution in 2 categories normal versus abnormal

## Table S2 – Clinical outcomes according to RDI among the 244 patients who had received at least 3 cycles of trabectedin

|  | Primary analysis | | | Second sensitivity analysis | | |
| --- | --- | --- | --- | --- | --- | --- |
|  | **Total**  **(N=244)** | **RDI < 80 % (N=106)** | **RDI ≥ 80 %**  **(N=138)** | **Total**  **(N=244)** | **RDI < 80 %**  **(N=106)** | **RDI ≥ 80 %**  **(N=138)** |
|  | **Progression-free survival from start of trabectedin** | | | **Progression-free survival from the 4^th^ cycle of trabectedin  or equivalent** | | |
| Number of events | 238 | 103 | 135 | 238 | 103 | 135 |
| Median PFS in months (IC95%) | 6.9 (5.9-7.8) | 8.4 (7.0-9.3) | 5.9 (4.4-6.8) | 4.5 (3.5-5.4) | 5.5 (4.1-6.5) | 3.7 (2.2-4.8) |
| PFS at 6 months (IC95%) | 56.2% (49.7-62.2) | 65.7% (55.8-73.9) | 48.9% (40.3-57.0) | 37.8% (31.6-43.7) | 44.8% (35.1-54.0) | 32.2% (24.5-40.1) |
| PFS at 12 months (IC95%) | 22.7% (17.7-28.2) | 27.6% (19.5-36.4) | 19.0% (12.9-26.0) | 16.6% (12.2-21.5) | 18.1% (11.5-26.0) | 15.4% (9.9-21.9) |
| PFS at 2 years (IC95%) | 5.8% (3.3-9.3) | 5.1% (1.9-10.6) | 6.3% (3.0-11.3) | 5.3% (2.9-8.7) | 5.1% (1.9-10.7) | 5.5% (2.5-10.3) |
| PFS at 3 years (IC95%) | 3.9% (1.9-7.0) | 3.8% (1.2-9.1) | 3.9% (1.5-7.3) | 3.4% (1.5-6.4) | 3.8% (1.2-9.2) | 3.2% (1.1-7.3) |
| PFS at 5 years (IC95%) | 2.4% (0.9-5.1) | 1.3% (0.1-5.9) | 3.2% (1.1-7.3) | 2.4% (0.9-5.2) | 1.3% (0.1-5.9) | 3.2% (1.1-7.3) |
|  | **Overall survival from start of trabectedin** | | | **Overall survival from the 4^th^ cycle of trabectedin  or equivalent** | | |
| Number of events | 219 | 96 | 123 | 219 | 96 | 123 |
| Median overall survival (IC95%) | 17.0 (15.1-20.0) | 18.2 (15.6-23.4) | 15.8 (13.2-19.7) | 14.5 (12.5-17.6) | 15.4 (12.5-19.9) | 13.7 (11.1-17.7) |
| OS at 1 year (IC95%) | 67.7% (61.4-73.2) | 72.4% (62.8-79.9) | 64.0% (55.4-71.5) | 57.6% (51.1-63.6) | 62.7% (52.7-71.2) | 53.7% (45.0-61.7) |
| OS at 2 year (IC95%) | 37.2% (31.0-43.3) | 38.3% (29.0-47.6) | 36.3% (28.2-44.4) | 32.7% (26.8-38.7) | 33.3% (24.4-42.4) | 32.2% (24.5-40.3) |
| OS at 3 year (IC95%) | 20.7% (15.7-26.2) | 21.9% (14.4-30.4) | 19.8% (13.4-27.2) | 18.0% (13.3-23.3) | 18.8% (11.8-27.0) | 17.4% (11.3-24.5) |
| OS at 5 year (IC95%) | 9.9% (6.3-14.4) | 11.0% (5.7-18.3) | 9.0% (4.7-15.2) | 9.9% (6.3-14.4) | 11.0% (5.7-18.3) | 9.0% (4.7-15.2) |
